# Effects of Internet Hospital Consultations on Psychological Burdens and Disease Knowledge During the Early Outbreak of COVID-19 in China: Cross-Sectional Survey Study

**DOI:** 10.2196/19551

**Published:** 2020-08-04

**Authors:** Lin Li, Gang Liu, Weiguo Xu, Yun Zhang, Mei He

**Affiliations:** 1 Department of Respiratory and Critical Care Medicine Mianyang Central Hospital Mianyang China; 2 Department of Orthopedics Mianyang Central Hospital Mianyang China; 3 Department of Neurology Mianyang Central Hospital Mianyang China; 4 Department of President’s Office Mianyang Central Hospital Mianyang China

**Keywords:** internet hospital, telemedicine, novel coronavirus disease, pandemic, psychological burden, disease cognition, coronavirus, COVID-19, public health, infectious disease

## Abstract

**Background:**

Coronavirus disease (COVID-19) has become a global threat to human health. Internet hospitals have emerged as a critical technology to bring epidemic-related web-based services and medical support to the public. However, only a few very recent scientific literature reports have explored the effects of internet hospitals on psychological burden and disease knowledge in major public health emergencies such as the COVID-19 pandemic.

**Objective:**

The aim of this study was to explore the role of internet hospitals in relieving psychological burden and increasing disease knowledge during the early outbreak of the COVID-19 pandemic.

**Methods:**

This survey was conducted from January 26 to February 1, 2020, during the early outbreak of COVID-19 in China. The platform used for the consultation was the WeChat public account of our hospital. To participate in the study, the patient was required to answer a list of questions to exclude the possibility of COVID-19 infection and confirm their willingness to participate voluntarily. Next, the participant was directed to complete the self-report questionnaire. After the internet consultation, the participant was directed to complete the self-report questionnaire again. The questionnaire included sections on general information, the General Health Questionnaire-28 (GHQ-28), and the participant’s worries, disease knowledge, and need for hospital treatment.

**Results:**

The total number of internet consultations was 4120. The consultation topics mainly included respiratory symptoms such as cough, expectoration, and fever (2489/4120, 60.4%) and disease knowledge, anxiety, and fear (1023/4120, 24.8%). A total of 1530 people filled out the questionnaires before and after the internet consultation. Of these people, 1398/1530 (91.4%) experienced psychological stress before the internet consultation, which significantly decreased after consultation (260/1530, 17.0%) (χ^2^_1_=1704.8, *P*<.001). There was no significant difference in the number of people who expressed concern about the COVID-19 pandemic before and after the internet consultation (χ^2^_1_=0.7, *P*=.43). However, the degree of concern after the internet consultation was significantly alleviated (*t_2699_*=90.638, *P*<.001). The main worries before and after consultation were the dangers posed by the disease and the risk of infection of family members. The scores of the self-assessment risk after the internet consultation were significantly lower than those before consultation (*t_3058_*=95.694, *P*<.001). After the consultation, the participants’ knowledge of the symptoms, transmission routes, and preventive measures of COVID-19 was significantly higher than before the consultation (*t_3058_*=–106.105, –80.456, and –152.605, respectively; all *P*<.001). The hospital treatment need score after the internet consultation decreased from 3.3 (SD 1.2) to 1.6 (SD 0.8), and the difference was statistically significant (*t_3058_*=45.765, *P*<.001).

**Conclusions:**

During the early outbreak of COVID-19, internet hospitals could help relieve psychological burdens and increase disease awareness through timely and rapid spread of knowledge regarding COVID-19 prevention and control. Internet hospitals should be an important aspect of a new medical model in public health emergency systems.

## Introduction

From severe acute respiratory syndrome (SARS) and H_1_N_1_ influenza to Middle East respiratory syndrome (MERS) and the latest new coronaviruses, new infectious diseases have become a global problem that seriously threatens human health [1,2]. In contrast to other viruses, severe acute respiratory syndrome coronavirus 2 (SARS-CoV-2), which causes coronavirus disease (COVID-19), has the characteristics of a long incubation period and strong infectivity [3,4]. On January 30, 2020, the World Health Organization declared the COVID-19 outbreak to be a public health emergency of international concern; at this point, all regions of China had reported cases of infection [5]. Soon, COVID-19 began spreading worldwide. The panic caused by this worldwide public health emergency has far surpassed those caused by the Middle East respiratory syndrome coronavirus (MERS-CoV) and severe acute respiratory syndrome coronavirus (SARS-CoV) pandemics [6]. Previous research has revealed a wide and profound range of impacts on people at the individual, community, and international levels during outbreaks of infection [7]. Because winter and spring are the peak seasons of influenza, people who have symptoms of upper respiratory infection, such as fever, fatigue, and cough, often fear that they are infected with COVID-19 [8]. If many people rush to the hospital in a short time, medical resources will be occupied and potential cross-infection and other problems will occur. Therefore, medical support at this time is essential. To help people seek timely professional medical advice and accurately guide them on when and how to report to a hospital, as well as to reduce the risk of cross-infection from population flow, the Chinese government has issued decrees that require domestic internet hospitals to make full use of the advantages of telemedicine to provide convenient and high-quality services in response to the epidemic [9,10]. Therefore, our hospital quickly responded and immediately set up free COVID-19 consultation services as the main form of telemedicine for the public during the epidemic. This action attracted widespread attention from all walks of life. Our internet hospital received more than 35,000 hits on in the first day, and the number of free medical consultations exceeded 1000 [11]. Internet hospitals, as a 21st-century approach to triage, enable efficient screening of patients and protect patients, clinicians, and the community from exposure during an infectious public health emergency [12]. However, only a few very recent scientific literature reports have explored the effects of internet hospitals on psychological burden and disease knowledge in major public health emergencies such as the COVID-19 pandemic. Thus, in addition to discussing symptoms and distinguishing disease, the clinicians also provide disease knowledge as well as medical or emotional support and understanding during internet consultation. A structured questionnaire containing questions regarding patients’ demographics, psychological state, and disease knowledge was used to explore the changes in these items before and after consultation. The goal of this research was to explore the role of internet hospitals in relieving psychological burden and increasing disease knowledge during the early outbreak of COVID-19 in China.

## Methods

### Participants

This survey was conducted from January 26 to February 1, 2020, which was the early period of the COVID-19 outbreak in China. The questionnaire was distributed as an electronic form that was collected and analyzed through the internet. Inclusion criteria were people who were aged ≥18 years and who completed the questionnaire. Exclusion criteria were people aged ≤17 years and who provided illogical responses to the questionnaire.

The Ethics Committee of Mianyang Central Hospital approved our study protocol and procedures of informed consent before the formal survey. Participants were required to answer a yes or no question to confirm their willingness to participate voluntarily. After confirmation of the question, the participant was directed to complete the self-report questionnaire.

### Internet Consultation Procedure

Before the internet hospital and web-based medical consultation system was set up, the project working group and engineers provided targeted training to the expert team, including the clinician-side use of internet hospitals, real-name registration, web-based consultation procedures, screening processes, and solutions to common problems. The online consultation was free to all. These free services could be obtained through the public WeChat account of our hospital by clicking Integrated Services, then clicking Internet Hospital, and finally clicking COVID-19 Consultation; then, real-time communication could be realized between clinicians and patients.

Free internet medical consultations for “fighting against COVID-19” could be accessed from 8 AM to 9 PM every day starting on January 26, 2020; clinicians talked with patients about their symptoms, helped them distinguish COVID-19 from the common cold, provided disease knowledge, and offered medical or emotional support and understanding.

The procedure of free COVID-19 consultation services was as follows. First, at the start of the conversation, the participant was required to answer a list of questions regarding their respiratory symptoms and provide detailed travel and exposure histories for screening. Participants who were not suspected of being infected with COVID-19 could proceed to the next step. Next, the participant was required to answer a yes or no question to confirm their willingness to participate voluntarily. After answering this question affirmatively, the participant was directed to complete the self-report questionnaire. Then, a clinician would communicate with the participant regarding their symptoms and psychological state, whether they had developed COVID-19, prevention and control of the disease, and any questions they had, until the participant actively ended the online consultation. Finally, the participant was directed to complete the self-report questionnaire again.

### Data Collection

The study instrument comprised a structured questionnaire packet including the following five sections.

#### 1. General Information

The general information section included questions about the respondent’s name, gender, age, education level, and occupation.

#### 2. Psychological State

The General Health Questionnaire-28 (GHQ-28) was used to evaluate the respondents’ psychological state. The GHQ-28 contains 28 items and is composed of four factors, namely physical condition, anxiety/insomnia, social dysfunction, and severe depression [13]. Each item is rated on a 4-level scale. The higher the total score, the greater the respondent’s psychological stress. A total GHQ-28 score of ≤5 points indicates that the subject has no psychological stress, 6-11 points indicates mild to moderate psychological stress, and >11 points indicates severe psychological stress.

#### 3. Concerns About COVID-19

This section contained questions regarding the respondents’ concerns about the COVID-19 pandemic, including whether they worried about the COVID-19 pandemic, and to what degree; they were also asked whether they mostly worried about the dangers of the disease, the risk of infection of family members and friends, isolation from their family and/or social environment, or the effects on their functional ability. Each item was evaluated by a dichotomy (yes or no) or a 9-level score from 1 (mild) to 9 (severe).

#### 4. Disease Knowledge

Questions about the respondents’ disease knowledge included their self-assessed risk, self-perceived seriousness of infection and difficulty of treatment, sufficiency of disease knowledge, and level of preparation for the epidemic. Each item was evaluated by a dichotomy (yes or no) or a 9-level score from 1 (high/disagree) to 9 (low/agree).

#### 5. Hospital Treatment Need

In this section, the respondents self-assessed their need to go to a hospital for further medical treatment. This need was also rated on a 9-level scale, from 1 (not needed) to 9 (needed).

The questionnaire administered before the internet consultation contained sections 1 to 5, and that administered after consultation contained sections 2 to 5.

### Statistical Analysis

The data were organized and analyzed using SPSS 25.0 software (IBM Corporation). Measurement data were expressed as mean (SD), and the independent sample *t* test was adopted for comparison between groups. For categorical data, frequencies and percentages were used, and the chi-square test was adopted for comparisons between groups. A two-tailed *P* value <.05 was considered statistically significant.

## Results

### Characteristics of the Participants

A total of 1530 people filled out the questionnaire before and after the internet consultation. The average age of the 1530 respondents was 38.9 years (SD 9.9); 998 (65.2%) were women and 532 (34.8%) were men. Among the 1530 respondents, 1101 (72.0%) were aged 18 to 40 years and 429 (28.0%) were older than 40 years. Regarding the respondents’ education levels, 96/1530 (6.3%) had a master’s degree or higher, 307/1530 (20.1%) had a bachelor’s degree, 678/1530 (44.3%) had a high school diploma, and 449/1530 (29.4%) had a junior high school education or below. Of the 1530 respondents, 212 (13.9%) were professionals (teacher/lawyer/student), 586 (38.3%) were employed in enterprises and institutions, 512 (33.5%) were laborers and service workers, and 220 (14.4%) had other occupations (retired, freelancer, or unemployed).

### General Information About the Internet Consultations

**Figure 1:**
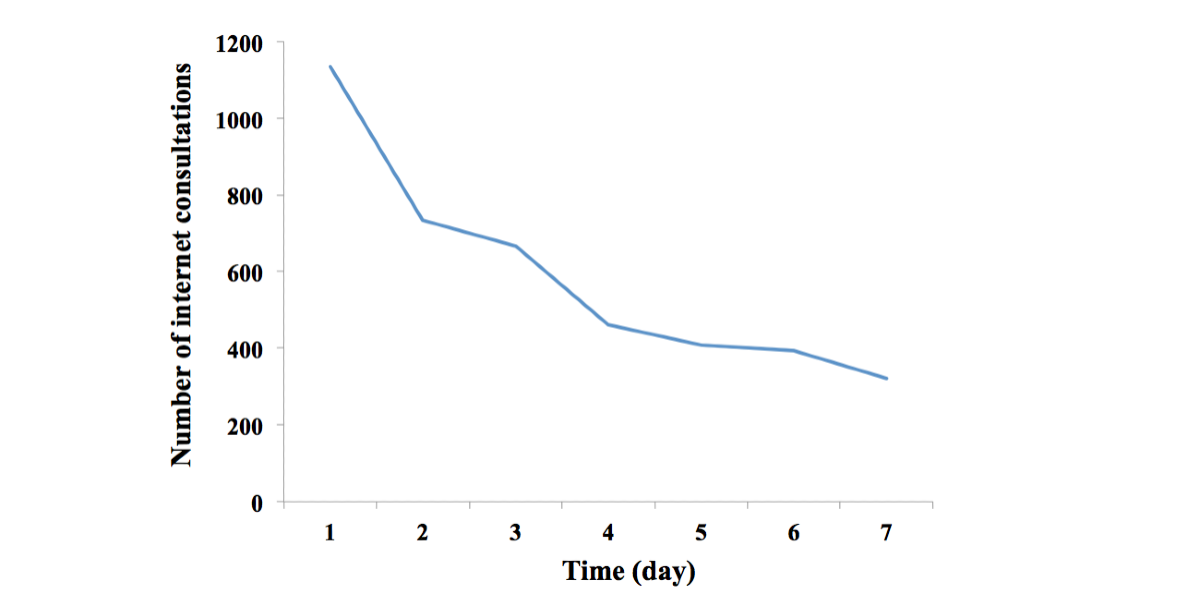
The change in the number of internet consultations with time.

A total of 4120 internet consultations were performed from January 26 to February 1, 2020. The number peaked on the first day and then gradually declined (Figure 1). The topics of discussion during the consultation mainly included respiratory symptoms such as cough, expectoration, and fever, accounting for 2489/4120 (60.4%) of the total consultations (Table 1). COVID-19 knowledge and anxiety and fear were the subjects of 1023/4120 consultations (24.8%) (Table 1). In the first 7 days, 5 people with fever and exposure histories were screened, and 136/1530 (8.9%) of the respondents needed to go to a hospital.

**Table 1:** Topics discussed during the internet consultations (N=4120).

Topic	n (%)
Respiratory symptoms, such as cough and expectoration	1628 (39.5)
Fever	861 (20.9)
COVID-19^a^ knowledge	598 (14.5)
Anxiety and fear	425 (10.3)
Consultation on chronic diseases	230 (5.6)
Gastrointestinal symptoms	155 (3.8)
Other	223 (5.4)

^a^COVID-19: coronavirus disease.

### Degrees of Psychological Stress Before and After the Internet Consultation

Before the internet consultation, 1398/1530 respondents (91.4%) reported experiencing psychological stress; this number was significantly higher than that after the internet consultation (260/1530, 17.0%), and the difference was statistically significant (χ^2^_1_=1704.8, *P*<.001). Among the 1530 respondents, 1352 (88.4%) scored 6 to 11 points on the GHQ-28, indicating light to moderate psychological stress; meanwhile, 46 (3.0%) scored >11 points on the GHQ-28, indicating severe psychological stress. The number of people with mild to moderate psychological stress before the internet consultation was significantly higher than that after consultation (χ^2^_1_=1693.8, *P*<.001). There was no statistically significant difference in the number of people with severe psychological stress before and after the internet consultation (*P*>.99) (Table 2).

**Table 2:** Degrees of psychological stress before and after the internet consultation (N=1530).

General Health Questionnaire-28 score (points)	Degree of psychological stress	Before internet consultation, n (%)	After internet consultation, n (%)	Chi-square (*df*=1)	*P* value
≤5	None	132 (8.6)	1270 (83.0)	1704.8	<.001
6-11	Mild to moderate	1352 (88.4)	214 (14.0)	1693.8	<.001
>11	Severe	46 (3.0)	46 (3.0)	<0.001	>.99

### Concerns About the COVID-19 Pandemic Before and After the Internet Consultation

There was no significant difference in the percentage of people who expressed concern about the COVID-19 pandemic before and after the internet consultation (χ^2^_1_=0.7, *P*=.43). However, the degree of concern after the internet consultation was significantly alleviated compared to before the internet consultation (*t_2699_*=90.638, *P*<.001). The main worries before and after the consultation were the dangers of the disease and the risk of infection of family members and friends. The respondents expressed fewer concerns about isolation from their family or social environment and effects on their functional ability. After the internet consultation, there were fewer concerns expressed about the dangers of the disease and the risk of infection of family members and friends than before the consultation, and the difference was statistically significant (χ^2^_1_=227.4 and 59.4, respectively; all *P*<.001) (Table 3).

**Table 3:** Concerns about the COVID-19 pandemic before and after the internet consultation (N=1530).

Question		Before internet consultation	After internet consultation	Comparison between groups	*P* value
I worry about the COVID-19^a^ pandemic, n (%)		1343 (87.8)	1358 (88.8)	χ^2^_1_=0.7	.43
Degree of worry (1=mild, 9=severe), mean (SD)		8.0 (0.9)	3.7 (1.5)	*t_2699_*=90.638	<.001
**Main cause of concern, n (%)**					
	The dangers of the disease	1122 (73.3)	668 (43.7)	χ^2^_1_=227.4	<.001
	The risk of infection of family and friends	948 (62.0)	736 (48.1)	χ^2^_1_=59.4	<.001
	Isolation from family and/or my social environment	194 (12.7)	171 (11.2)	χ^2^_1_=1.6	.22
	The effects on my functional ability	200 (13.1)	168 (11.0)	χ^2^_1_=3.2	.09

^a^COVID-19: coronavirus disease.

### Disease Knowledge Before and After the Internet Consultation

The self-assessment risk scores before the internet consultation were significantly higher than those after the consultation (*t_3058_*=95.694, *P*<.001). There was no statistically significant difference in the scores of the self-perceived seriousness of infection and difficulty of treatment before and after the internet consultation (*t_3058_*=1.333, *P*=.18 and *t_3058_*=0.242, *P*=.81). After consultation, the respondents’ knowledge of the symptoms, transmission routes, and preventive measures of COVID-19 was higher than that before consultation, and the difference was statistically significant (*t_3058_*=–106.105, –80.456, and –152.605, respectively; all *P*<.001). In terms of disease treatment and prognosis, the levels of knowledge were relatively low both before and after the internet consultation, and the respondents all reported that they were not prepared to fight the epidemic (*P*=.07) (Table 4).

**Table 4:** Respondents’ knowledge regarding COVID-19 before and after the internet consultation.

Question		Before internet consultation, mean (SD)	After internet consultation, mean (SD)	*t_3058_*	*P* value
Self-assessment risk score (1=low, 9=high)		8.1 (0.9)	4.2 ± 1.3	95.694	<.001
I think infection may seriously impair my health (1=disagree, 9=agree)		5.1 (1.2)	5.1 (1.0)	1.333	.18
I think COVID-19^a^ is hard to cure after infection (1=disagree, 9=agree)		4.6 (1.1)	4.6 (1.3)	0.242	.81
**I think I have sufficient information about (1=disagree, 9=agree):**					
	Symptoms	3.7 (1.1)	7.8 (1.0)	–106.105	<.001
	Treatment	3.2 (1.1)	3.2 (1.2)	–0.765	.45
	Prognosis	2.9 (1.0)	2.9 (1.4)	–1.579	.11
	Transmission routes	4.7 (1.0)	7.7 (1.0)	–80.456	<.001
	Preventive measures	2.9 (0.9)	8.0 (0.9)	–152.605	<.001
I think I am prepared to fight the epidemic (1=disagree, 9=agree)		3.0 (1.5)	3.1 (1.4)	–1.799	.07

^a^COVID-19: coronavirus disease

### Need for Hospital Treatment Before and After the Internet Consultation

The score of hospital treatment need after the internet consultation was 1.6 (SD 0.8), which is lower than that before the consultation (3.3, SD 1.2); the difference was statistically significant (*t_3058_*=45.765, *P*<.001).

## Discussion

### Principal Findings

Our results showed that after the internet consultation, the number of respondents who had psychological stress reactions decreased and their degrees of concern about the COVID-19 pandemic were alleviated. Additionally, the respondents’ knowledge of the symptoms, transmission routes, and preventive measures of COVID-19 increased after the internet consultation. Our results revealed the important role of internet hospitals in the response to infectious public health emergencies through their timely and rapid spread of knowledge regarding prevention and control of COVID-19.

In recent years, our hospital has relied on the “internet + medical care” model of our internet hospital to provide people with web-based consultations, appointments, electronic prescriptions, and other services, achieving a new mode of internet medical care with a combination of online and offline medical services. Since the outbreak of the epidemic, our hospital has quickly established a multidisciplinary project working group led by the director of the hospital and senior medical experts, including physicians, nursing experts, psychologists, and health managers [11]. Regulations were established to ensure the quality and standardization of internet consultation services, including a “Notice on the Standardized Implementation of Internet COVID-19 Consultation” and “Operating Manual for COVID-19 Online Consultation.” Over 7 days, from January 26 to February 1, 2020, the total number of online consultations in the internet hospital was 4120. Among these, 2489/4120 people (60.4%) consulted on respiratory symptoms such as cough, sputum, and fever, 598/4120 (14.5%) on knowledge of COVID-19, and 425/4120 (10.3%) on psychological problems related to anxiety and fear. In addition to helping to distinguish the common cold, the symptoms of which are similar to early signs of COVID-19, we noted that consultation on the knowledge of COVID-19 and psychological problems constituted nearly 25% of the topics discussed (1023/4120 consultations, 24.8%). This showed that during the epidemic, people have a strong need for disease knowledge and a high risk of psychological stress, which provides good opportunities for telemedicine.

The results of the study showed that during the early period of the outbreak, 1343/1530 (87.8%) of people before the internet consultation and 1358/1530 (88.8%) after the internet consultation expressed concern about the COVID-19 pandemic; however the degrees of concern before and after the consultation were different. The degree of concern before the consultation was generally more severe. We also found that the number of people with psychological stress or mild to moderate psychological stress before internet consultation was significantly higher than that after consultation. This was consistent with the fact that these respondents believed that the risk of disease was high before the online consultation. Thus, it can be seen that in major public health emergencies such as the COVID-19 pandemic, internet hospitals enable people to obtain timely disease and symptom consultations, gain knowledge regarding the prevention and control of COVID-19, learn about best practices during home quarantine, and receive prompt responses to their concerns [14]. All these benefits will be propitious to reduce their psychological stress and fear about the epidemic.

The outbreak of COVID-19 occurred at the Lunar New Year in China. If we had failed to raise awareness of COVID-19 and inform people about the correct prevention and control measures of the disease in the shortest time, the epidemic would probably have spread further [15]. At this time, the traditional methods of spreading knowledge about diseases, such as centralized training, lectures, and leaflet dissemination, are costly and inefficient; additionally, these methods are not sufficiently fast, and the coverage is narrow. Accordingly, the internet has become the best tool and information carrier for epidemic prevention [16]. Our research results showed that after the internet consultation, the respondents’ knowledge of the symptoms, transmission routes, and preventive measures of COVID-19 was higher than that before consultation. The respondents’ psychological stress and degree of concern about the epidemic were alleviated after the internet consultation, which may be related to their deeper understanding of the disease. It has been confirmed that it is feasible and effective to promptly spread knowledge regarding epidemic prevention and control through internet hospitals and to increase people’s knowledge of the disease.

In contrast to rescues in natural disasters, hospitals are obviously unable to meet the needs of all people during major public health emergencies such as the COVID-19 pandemic. Even hospitals are prone to cluster disease and virus spread [17]. Therefore, a decentralized medical assistance model is emphasized in public health emergencies, and the internet plays a key role similar to that of tiered medical service in this decentralization. After online consultation, only 136/1530 (8.89%) of the survey respondents needed to go to a hospital for further examination, and the score of hospital treatment need was also significantly lower than that before the consultation; this indicates that most online consultations are only due to the common cold or mild discomfort that can be monitored at home. Through internet consultations, these patients learn that they only require observation or can simply take nonprescription drugs. The web-based internet hospital service provided patients with necessary medical guidance during the epidemic and reduced the mobility of people to the hospital, which is extremely beneficial to cut off transmission routes and reduce person-to-person transmission [18,19].

### Limitations

One limitation of this study is that it is based on a web-based questionnaire. It is possible that the survey did not reach underdeveloped areas due to limited technology availability and that it omitted people who are not comfortable using the internet. Another limitation is that self-reported levels of psychological impact are not always aligned with assessment by mental health professionals. Irrespective of the above limitations, this study provides invaluable information on the role of internet hospitals on people during the early outbreak of COVID-19 in China. Most importantly, our findings directly demonstrate that internet hospitals are perfectly suited to this pandemic situation because they help relieve psychological burdens on patients and increase disease knowledge. These effects are crucial because ensuring that the general public is well-informed about a condition such as COVID-19 can reduce unnecessary psychological burdens without increasing risk of cross-infection.

With the increasingly powerful functions of the internet and handheld smart devices, internet hospitals are an increasingly feasible way to spread disease knowledge [20,21]. Internet hospitals should be an important part of the emergency system and a novel medical model, especially during major public health emergencies. These hospitals will be indispensable not only during the COVID-19 pandemic but also in future outbreaks of infection.

## Abbreviations

COVID-19: coronavirus disease
GHQ-28: General Health Questionnaire-28
MERS: Middle East respiratory syndrome
MERS-CoV: Middle East respiratory syndrome coronavirus
SARS: severe acute respiratory syndrome
SARS-CoV: severe acute respiratory syndrome coronavirus
SARS-CoV-2: severe acute respiratory syndrome coronavirus 2
